# Large Paratesticular Cellular Angiofibroma: Imaging Findings and Pathological Correlation

**DOI:** 10.7759/cureus.107981

**Published:** 2026-04-29

**Authors:** Shahd Mohammedain, Nuha Sabbagh, Sarah Sayed, Amal Al Rashid, Aalaa Kambal

**Affiliations:** 1 Radiology, Hamad Medical Corporation, Doha, QAT; 2 Pathology, Hamad Medical Corporation, Doha, QAT

**Keywords:** case report, cellular angiofibroma, mri, paratesticular tumor, scrotal mass

## Abstract

Cellular angiofibroma is a rare benign tumor that typically occurs in the genitourinary region and may be difficult to distinguish from other scrotal masses. We report a case of a 41-year-old man presenting with a slowly enlarging, painless scrotal swelling. Imaging demonstrated a well-defined extratesticular lesion without clear features of malignancy. The patient underwent surgical excision with preservation of surrounding structures. Histological evaluation confirmed the diagnosis of cellular angiofibroma, with no evidence of aggressive behavior. This case highlights the importance of considering this rare entity in the differential diagnosis of paratesticular masses and emphasizes the role of histopathology in establishing the diagnosis and guiding appropriate management.

## Introduction

Cellular angiofibroma (CAF) is a rare, benign mesenchymal tumor that predominantly arises in the subcutaneous soft tissues of the genitourinary region, with a predilection for the vulvoperineal area in women and the inguinoscrotal region in men [1,2]. While generally considered a neoplasm of superficial soft tissue, its manifestation in the paratesticular region (originating from the spermatic cord, epididymis, or tunica) is particularly uncommon [3].

The clinical presentation of a paratesticular mass, typically as a painless scrotal swelling, can mimic a wide array of more common entities, including inguinal hernia, spermatocele, or, critically, more aggressive spindle cell tumors such as sarcoma or malignant germ cell tumors [3,4]. This diagnostic challenge necessitates careful radiological and, ultimately, histopathological evaluation to ensure appropriate management, which is typically local surgical excision due to its benign nature [5].

Given the relative scarcity of paratesticular CAF, reporting individual cases remains valuable for augmenting the collective clinical and pathological understanding of this tumor [3]. We herein present a case of paratesticular CAF in a 41-year-old male to highlight its clinical presentation, radiological features, characteristic pathological findings, and successful management.

## Case presentation

A 41-year-old male with an unremarkable past medical and surgical history presented in June 2025 with a painless right hemiscrotal swelling that had been gradually increasing in size over a period of approximately two years. The patient denied any associated lower urinary tract symptoms (LUTS), hematuria, urethral discharge, dysuria, or history of trauma.

Clinical and imaging findings

Physical examination revealed a large, hard mass in the right hemiscrotum, accompanied by associated scrotal edema. The mass was separate from the palpably normal right testis.

**Figure 1:**
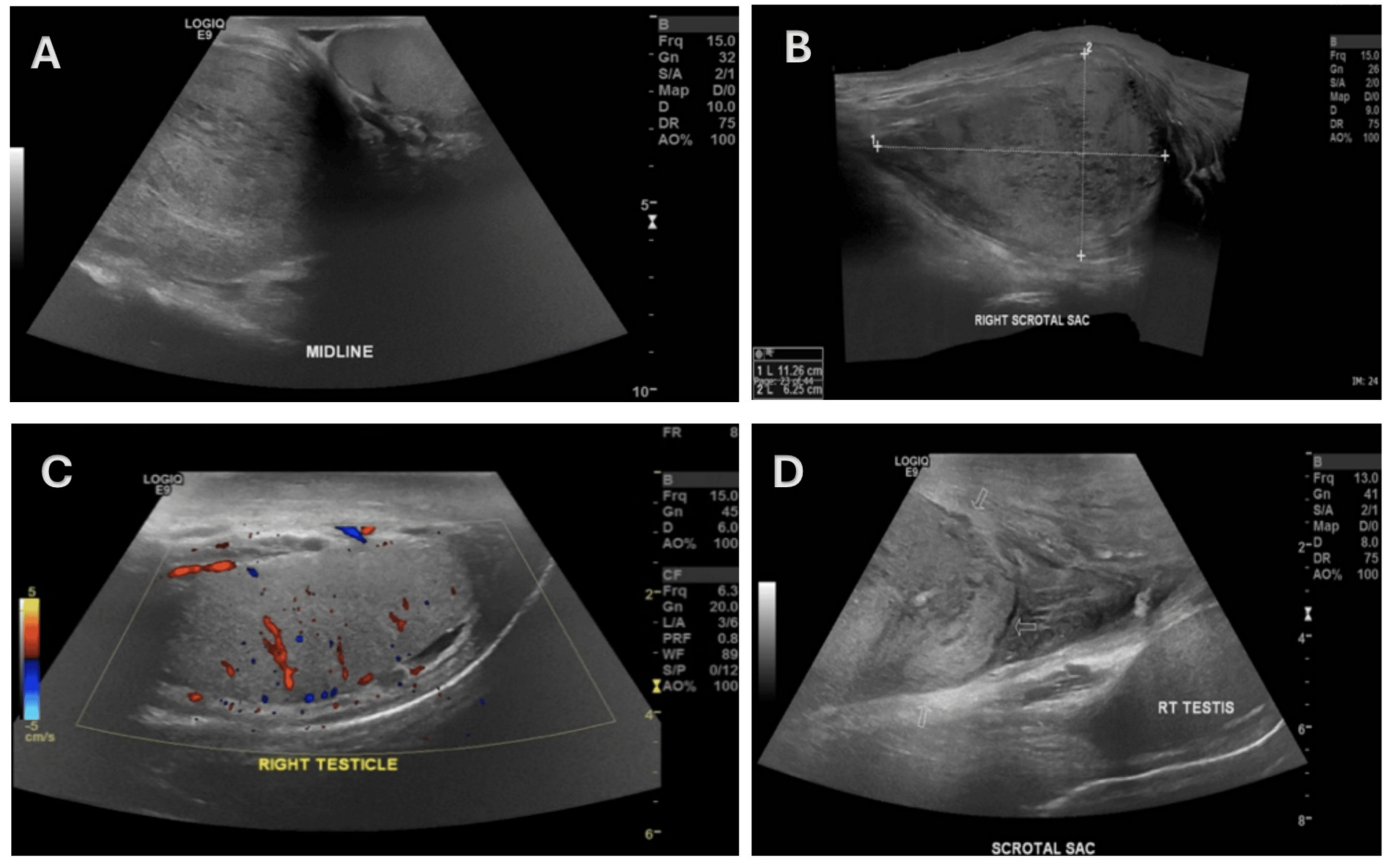
Scrotal ultrasound (A-B) Grayscale ultrasound images showing a large extratesticular lesion measuring 11 × 6 cm. (C) Doppler images demonstrating internal vascularity within the lesion. (D) Normal appearance of the right testes.

Initial scrotal ultrasound identified a large, well-circumscribed, heterogeneous, predominantly isoechoic extratesticular solid lesion measuring 11 cm in maximal dimension (Figure 1A-C), with internal vascularity on Doppler interrogation, highly suggestive of irreducible hernial content or a solid neoplasm. The right testis appeared normal (Figure 1D), although a right varicocele was noted. Tumor markers, including alpha-fetoprotein, human chorionic gonadotropin beta-subunit, and lactate dehydrogenase, were within normal limits.

**Figure 2:**
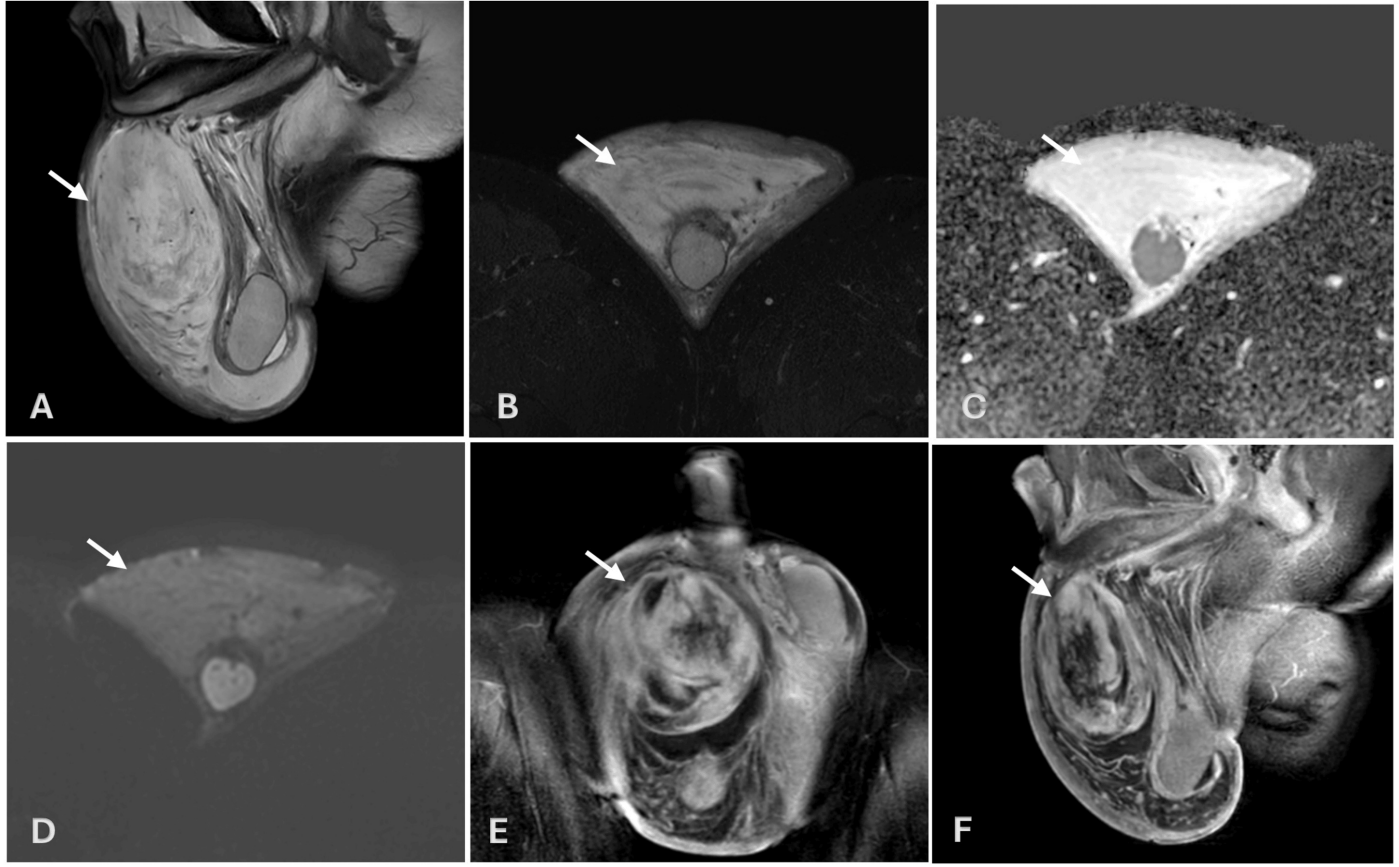
Scrotal MRI (A) T2-weighted image demonstrates a hyperintense extratesticular lesion (white arrow) with no significant signal drop on the T2 fat-saturated sequence (B). Diffusion-weighted imaging (C) and the corresponding apparent diffusion coefficient map (D) show no evidence of diffusion restriction within the lesion. Postcontrast images (E, F) demonstrate heterogeneous enhancement of the lesion.

MRI of the scrotum confirmed the presence of a right extratesticular mass measuring 10 × 9 cm. The mass was abutting or inseparable from the spermatic cord and tunica, exhibiting hyperintense T2 signal (Figure 2A) and heterogeneous postcontrast enhancement (Figure 2E,F). No significant diffusion restriction or testicular invasion was observed (Figure 2C,D), although the right testis was mildly smaller compared to the left. The scrotal wall was significantly thickened and edematous. The radiological impression favored a mesenchymal tumor, with CAF or sarcoma as the differential diagnoses. 

Surgical course

The patient underwent right inguinal exploration under spinal anesthesia, with testis-sparing excision of the paratesticular mass. The lesion was carefully dissected from the surrounding scrotal tissues and separated from the spermatic cord and testis, with preservation of the tunica vaginalis. The mass was excised completely without orchiectomy, and the specimen was submitted for histopathological analysis.

The patient was reviewed in the outpatient clinic two weeks postoperatively and had an uneventful recovery, with satisfactory wound healing and no immediate postoperative complications. Following histopathological confirmation of a benign CAF, no further scheduled follow-up was deemed necessary.

Pathological diagnosis

The histological features of CAF are distinct, characterized by a well-circumscribed, highly cellular proliferation of bland, spindle-shaped cells interspersed with numerous small- to medium-sized blood vessels, often thick-walled and hyalinized, along with ropey collagen bundles [2,5].

**Figure 3:**
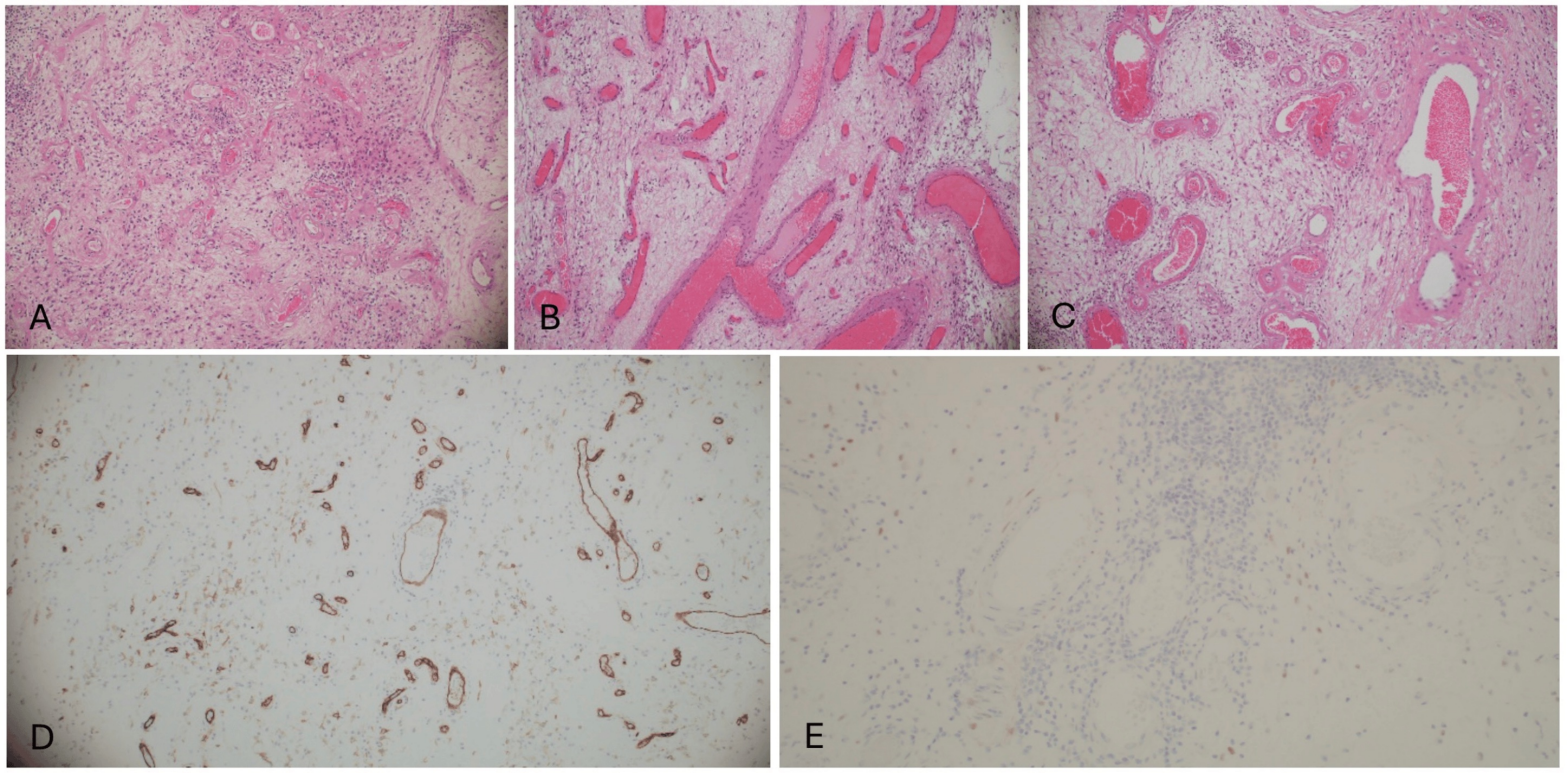
Histopathology Microscopic examination demonstrates a well-circumscribed tumor composed of spindle cells and small- to medium-sized blood vessels with hyalinized walls. The lesion shows low-to-moderate stromal cellularity, abundant collagenous stroma, and a moderate chronic perivascular lymphocytic inflammatory infiltrate (A–C). Immunohistochemical studies show weak positivity for CD34 (D) and focal positivity for estrogen receptor (ER) (E).

Microscopic examination revealed a well-circumscribed tumor composed of spindle cells and small- to medium-sized blood vessels with hyalinized walls (Figure 3A-C). The lesion featured low to moderate stromal cellularity, abundant collagenous stroma, and a moderate chronic perivascular lymphocytic inflammatory infiltrate (Figure 3A-C). There was no evidence of myoid differentiation, increased mitotic activity, or infiltration of soft tissue.

Immunohistochemical studies showed focal positivity for estrogen receptor (ER) (Figure 3E) and weak positivity for CD34 (Figure 3D), which is characteristic. The lesion was negative for markers of malignancy and other tumor types (e.g., CKAE1/AE3, S100, HMB45). 

The final diagnosis, based on morphological and immunohistochemical features, was a CAF of the paratesticular region. No features of malignancy were identified.

## Discussion

Paratesticular CAF is an uncommon benign mesenchymal tumor, and its diagnosis remains challenging because of its significant overlap in clinical and imaging characteristics with other extratesticular lesions. Although CAF most frequently arises in the inguinoscrotal region of men, only a limited number of well-documented paratesticular cases exist in the literature [1-5]. The typical clinical presentation of a slowly enlarging, painless scrotal mass, as in our patient, provides little diagnostic specificity, as similar findings occur with inguinal hernias, adenomatoid tumors, lipomas, leiomyomas, and even soft-tissue sarcomas [4,6].

Radiologic considerations

Ultrasound is the standard initial imaging modality for scrotal masses, but its ability to differentiate CAF from other soft tissue tumors is limited. Most CAFs appear as well-defined, solid, hypoechoic extratesticular masses, findings that can be indistinguishable from more aggressive spindle cell tumors [4,6]. In our case, ultrasound demonstrated a large extratesticular mass without defining features favoring a benign lesion.

MRI has been shown to better characterize the internal architecture of CAFs because of its sensitivity to tissue composition. CAFs typically exhibit intermediate-to-high T2 signal intensity, reflecting varying degrees of cellularity, vascularity, and fibrous stroma, often with heterogeneous enhancement after contrast administration [5,7]. The absence of significant diffusion restriction on DWI, observed in our patient, is consistent with the benign nature of CAF and has been reported in similar cases [3,5]. However, MRI cannot reliably exclude low-grade sarcoma, especially in large or atypical masses, necessitating histopathological correlation for definitive diagnosis.

Histopathologic and immunohistochemical features

Microscopically, CAFs demonstrate a characteristic combination of bland spindle cells, thick-walled vessels with hyalinized walls, and collagenous stroma, often with scattered inflammatory infiltrates [1,2]. Immunohistochemically, CAFs typically express CD34 and variably express estrogen and progesterone receptors, supporting their origin from hormonally responsive mesenchymal cells [1,8]. Our case mirrored these features, showing CD34 positivity and focal ER expression. Importantly, the absence of atypia, high mitotic activity, necrosis, or infiltrative growth favors a benign diagnosis.

Differential diagnosis

From an imaging perspective, the differential diagnosis of paratesticular CAF includes spindle cell lipoma, aggressive angiomyxoma, solitary fibrous tumor, and leiomyomatous tumors. Spindle cell lipoma may appear as a well-circumscribed lesion with internal fat components, unlike CAF, which is typically more fibrous and vascular [2,8]. Aggressive angiomyxoma often demonstrates a more infiltrative appearance, with marked T2 hyperintensity and a characteristic layered or swirling enhancement pattern on MRI [9]. Solitary fibrous tumors may show avid enhancement related to hypervascularity and can demonstrate lower T2 signal due to greater fibrous content [10]. Leiomyoma and leiomyosarcoma may appear as solid enhancing masses, although leiomyosarcoma more often shows invasive features, heterogeneity, necrosis, or diffusion restriction suggestive of malignancy [4,6]. Recognition of these imaging distinctions may help narrow the differential diagnosis, although histopathologic confirmation remains essential.

Management and prognosis

Complete local excision remains the treatment of choice. While some authors advocate testis-sparing surgery when feasible, many cases, including ours, require inguinal exploration to adequately assess tumor extent and ensure complete resection. CAFs have an excellent prognosis and exceedingly low recurrence rates, even in tumors showing atypical or "bizarre" nuclear features [8,11]. Although recurrence is exceedingly uncommon, clinical follow-up may be considered on an individualized basis.

To our knowledge, this represents one of the largest reported paratesticular CAFs with nonaggressive imaging characteristics, emphasizing that tumor size alone does not reliably predict malignancy in paratesticular lesions. Given the large size of the mass in the current case (approximately 10-11 cm), the benign pathology underscores an important radiologic learning point.

## Conclusions

Paratesticular CAF is a rare benign mesenchymal tumor that can closely mimic other more common or aggressive paratesticular masses clinically and radiologically. Although ultrasound and MRI contribute important diagnostic clues, such as well-circumscribed margins, heterogeneous T2 hyperintensity, lack of diffusion restriction, and variable enhancement, definitive diagnosis requires histopathology and immunohistochemistry. Surgical excision is curative in most patients, and prognosis is excellent, with recurrence being exceedingly uncommon. Increased recognition of CAF and its imaging features is essential to ensure appropriate surgical management.
